# Double-blind, randomized, controlled, trial to assess the efficacy of allogenic mesenchymal stromal cells in patients with acute respiratory distress syndrome due to COVID-19 (COVID-AT): A structured summary of a study protocol for a randomised controlled trial

**DOI:** 10.1186/s13063-020-04964-1

**Published:** 2021-01-06

**Authors:** Concepción Payares-Herrera, María E. Martínez-Muñoz, Inés Lipperheide Vallhonrat, Rosa Malo de Molina, Manuel Pérez Torres, Andrea Trisan, Isabel Salcedo de Diego, Rosalía Alonso, Rocío Zafra, Trinidad Donaire, Rocío Sánchez, Juan José Rubio, Rafael F. Duarte Palomino, Cristina Avendaño Solá

**Affiliations:** 1 Department of Clinical Pharmacology, Hospital Universitario Puerta de Hierro, Madrid, Majadahonda Spain; 2 Department of Haematology and Cell Production Unit, Hospital Universitario Puerta de Hierro, Madrid, Majadahonda Spain; 3 Department of Intensive Care Medicine, Hospital Universitario Puerta de Hierro, Madrid, Majadahonda Spain; 4 Department of Respiratory Medicine, Hospital Universitario Puerta de Hierro, Madrid, Majadahonda Spain

**Keywords:** COVID-19, randomised controlled trial, protocol, acute respiratory distress syndrome, mesenchymal stromal cells

## Abstract

**Objectives:**

1. To assess the efficacy of Mesenchymal Stromal Cells (MSC) versus a control arm as described in the primary endpoint.

2. To evaluate the effects of MSC on the secondary efficacy endpoints.

3. To evaluate the safety and tolerability profiles of MSC.

4. To study soluble and cellular biomarkers that might be involved in the course of the disease and the response to the investigational product.

**Trial design:**

A double-blind, randomized, controlled, trial to evaluate the efficacy and safety of MSC intravenous administration in patients with COVID-induced Acute Respiratory Distress Syndrome (ARDS) compared to a control arm.

**Participants:**

The trial is being conducted at a third level hospital, Hospital Universitario Puerta de Hierro, in Majadahonda, Madrid (Spain).

**Inclusion criteria**

1. Informed consent prior to performing study procedures (witnessed oral consent with written consent by representatives will be accepted to avoid paper handling). Written consent by patient or representatives will be obtained whenever possible.

2. Adult patients ≥18 years of age at the time of enrolment.

3. Laboratory-confirmed SARS-CoV-2 infection as determined by Polymerase Chain Reaction (PCR), in oropharyngeal swabs or any other relevant specimen obtained during the course of the disease. Alternative tests (e.g., rapid antigen tests) are also acceptable as laboratory confirmation if their specificity has been accepted by the Sponsor.

4. Moderate to severe ARDS (PaO2/FiO2 ratio equal or less than 200 mmHg) for less than 96 hours at the time of randomization.

5. Patients requiring invasive ventilation are eligible within 72 hours from intubation.

6. Eligible for ICU admission, according to the clinical team.

**Exclusion criteria**

1. Imminent and unavoidable progression to death within 24 hours, irrespective of the provision of treatments (in the opinion of the clinical team).

2. “Do Not Attempt Resuscitation” order in place.

3. Any end-stage organ disease or condition, which in the investigator’s opinion, makes the patient an unsuitable candidate for treatment.

4. History of a moderate/severe lung disorder requiring home-based oxygen therapy.

5. Patient requiring Extracorporeal Membrane Oxygenation (ECMO), haemodialysis or hemofiltration at the time of treatment administration.

6. Current diagnosis of pulmonary embolism.

7. Active neoplasm, except carcinoma in situ or basalioma.

8. Known allergy to the products involved in the allogeneic MSC production process.

9. Current pregnancy or lactation (women with childbearing potential should have a negative pregnancy test result at the time of study enrolment).

10. Current participation in a clinical trial with an experimental treatment for COVID-19 (the use of any off-label medicine according to local treatment protocols is not an exclusion criteria).

11. Any circumstances that in the investigator’s opinion compromises the patient’s ability to participate in the clinical trial.

**Intervention and comparator:**

- Experimental treatment arm: Allogeneic MSC (approximately 1 x 10^6^ cells/kg).

- Control arm: placebo solution (same composition as the experimental treatment, without the MSC).

One single intravenous dose of the assigned treatment will be administered on Day 0 of the study.

All trial participants will receive standard of care (SOC). In the context of the current worldwide pandemic, SOC can include medicines that are being used in clinical practice (e.g. lopinavir/ritonavir; hydroxy/chloroquine, tocilizumab, etc.), as well as those authorised for COVID (e.g., remdesivir).

**Main outcomes:**

**Primary endpoint:**

Change in the PaO2/FiO2 ratio from baseline to day 7 of treatment administration, or to the last available PaO2/FiO2 ratio if death occurs before day 7.

**Secondary endpoints:**

- All-cause mortality on days 7, 14, and 28 after treatment.

- PaO2/FiO2 ratio at baseline and days 2, 4, 7, 14 and 28 after treatment.

- Oxygen saturation (by standardized measurement) at baseline, daily until day 14, and on day 28 after treatment.

- Time to PaO2/FiO2 ratio greater than 200 mmHg.

- Subjects’ clinical status on the WHO 7-point ordinal scale at baseline, daily until day 14, and on day 28 after treatment.

- Time to an improvement of one category from admission on the WHO 7-point ordinal scale.

- Percentage of patients that worsen at least one category on the WHO 7-point ordinal scale.

- Percentage of patients that improve at least one category (maintained 48h) on the WHO 7-point ordinal scale.

- Sequential Organ Failure Assessment (SOFA) scale at baseline and days 2, 4, 7, 14 and 28 after treatment.

- Duration of hospitalization (days).

- Duration of ICU stay (days).

- Oxygen therapy-free days in the first 28 days after treatment.

- Duration of supplemental oxygen.

- Incidence of and duration of non-invasive and invasive mechanical ventilation in the first 28 days after treatment.

- Mechanical ventilation-free days in the first 28 days after treatment.

- Ventilation parameters.

- Incidence of new onset pulmonary fibrosis at 3 and 12 months after treatment, based on CT scan and pulmonary function tests.

- Survival at 3 and 12 months.

- Cumulative incidence of Serious Adverse events (SAEs) and Grade 3 and 4 Adverse Events (AEs).

- Cumulative incidence of Adverse Drug Reactions (ADR) in the experimental treatment arm.

- Cumulative incidence of AEs of special interest.

- Levels of analytical markers (C-Reactive Protein, lymphocyte and neutrophil counts, lymphocyte subpopulations, LDH, ferritin, D-dimer, coagulation tests and cytokines...) at baseline and days 2, 4, 7, 14 and 28 after treatment.

- Other soluble and cellular biomarkers that might be involved in the course of the disease and the response to MSC.

**Randomisation:**

The assignment to treatment will be carried out randomly and blinded, with a 1:1 allocation. Randomization will be done through a centralized system embedded in the electronic Case Report Form (CRF).

**Blinding (masking):**

To ensure blinding, treatments will be prepared for administration at the Cell Production Unit and the administration of the treatment will be masked, not allowing the identification of the Investigational Medicinal Product (IMP).

**Numbers to be randomised (sample size):**

A total of 20 participants are planned to be randomized, 10 to each treatment group.

**Trial Status:**

Protocol version: 1.2, dated October 14th, 2020

Start of recruitment: 01/10/2020

End of recruitment (estimated): December 2020.

**Trial registration:**

EudraCT Number: 2020-002193-27, registered on July 14^th^, 2020.

NCT number: NCT04615429, registered on November 4^th^, 2020.

**Full protocol:**

The full protocol is attached as an additional file, accessible from the Trials website (Additional file 1). In the interest in expediting dissemination of this material, the familiar formatting has been eliminated; this Letter serves as a summary of the key elements of the full protocol.

**Supplementary Information:**

The online version contains supplementary material available at 10.1186/s13063-020-04964-1.

## Supplementary Information

**Additional file 1.**

## Data Availability

Not applicable.

